# *Drosophila* F-BAR protein Syndapin contributes to coupling the plasma membrane and contractile ring in cytokinesis

**DOI:** 10.1098/rsob.130081

**Published:** 2013-08

**Authors:** Tetsuya Takeda, Iain M. Robinson, Matthew M. Savoian, John R. Griffiths, Anthony D. Whetton, Harvey T. McMahon, David M. Glover

**Affiliations:** 1 Department of Genetics, University of Cambridge, Downing Street, Cambridge CB2 3EH, UK; 2 Peninsula College of Medicine and Dentistry, University of Plymouth, Plymouth PL6 8BU, UK; 3 Faculty of Medical and Human Sciences, Manchester Academic Health Science Centre, Institute for Cancer Sciences, University of Manchester, Manchester M20 3LJ, UK; 4 MRC Laboratory of Molecular Biology, Francis Crick Avenue, Cambridge CB2 0QH, UK

**Keywords:** cytokinesis, F-BAR, actomyosin contractile ring, membrane, phosphorylation

## Abstract

Cytokinesis is a highly ordered cellular process driven by interactions between central spindle microtubules and the actomyosin contractile ring linked to the dynamic remodelling of the plasma membrane. The mechanisms responsible for reorganizing the plasma membrane at the cell equator and its coupling to the contractile ring in cytokinesis are poorly understood. We report here that Syndapin, a protein containing an F-BAR domain required for membrane curvature, contributes to the remodelling of the plasma membrane around the contractile ring for cytokinesis. Syndapin colocalizes with phosphatidylinositol 4,5-bisphosphate (PI(4,5)P_2_) at the cleavage furrow, where it directly interacts with a contractile ring component, Anillin. Accordingly, Anillin is mislocalized during cytokinesis in Syndapin mutants. Elevated or diminished expression of Syndapin leads to cytokinesis defects with abnormal cortical dynamics. The minimal segment of Syndapin, which is able to localize to the cleavage furrow and induce cytokinesis defects, is the F-BAR domain and its immediate C-terminal sequences. Phosphorylation of this region prevents this functional interaction, resulting in reduced ability of Syndapin to bind to and deform membranes. Thus, the dephosphorylated form of Syndapin mediates both remodelling of the plasma membrane and its proper coupling to the cytokinetic machinery.

## 2. Introduction

Cytokinesis is the final step of cell division required to partition the newly segregated daughter chromosomes, cytoplasmic macromolecules and organelles into daughter cells [1]. Defects in this process lead to aneuploidy associated with infertility, developmental defects and cancers [2,3]. Cytokinesis in most eukaryotes is accomplished through contraction of the contractile ring, which in turn leads to constriction of the plasma membrane [4]. Recent studies have shown that membrane trafficking and remodelling machineries also play crucial roles in both furrowing and abscission [5,6]. The processes whereby the plasma membrane is locally remodelled for cytokinesis and the nature of the molecular components that couple the membrane to the contractile ring are still uncertain. Anillin, an essential scaffold protein in cytokinesis [7], has been suggested to play a role as a molecular linker between the plasma membrane and the contractile ring [8,9], but it appears to be functionally redundant with other membrane anchor(s) that remain to be identified.

F-BAR proteins are evolutionarily conserved proteins that facilitate membrane curvature [10,11]. The membrane-binding F-BAR domains form dimeric positively charged modules that bind to negatively charged lipids (e.g. PI(4,5)P_2_). Most F-BAR proteins also interact with cytoskeletal regulators to provide a functional interface between membranes and the cytoskeleton in diverse processes, including endocytosis, cell motility and cell adhesion. The F-BAR proteins of fission yeast (Cdc15p) and budding yeast (Hof1p) are already known to play essential roles in cytokinesis [12,13]. Cdc15p regulates contractile ring assembly and maintenance [14,15] and organizes sterol-rich membrane domains during cytokinesis [16]. Hof1p is also required for regulation of the contractile ring dynamics and septum formation in cytokinesis [17,18]. In contrast with the yeasts, although the F-BAR proteins, mouse PSTPIP [19] and human Syndapin 2 [20], have been implicated in animal cell cytokinesis, their precise molecular function has not been defined.

Here, we provide direct evidence for a role of a *Drosophila* F-BAR protein, Syndapin, in cytokinesis. Syndapin is involved in multiple cellular processes, such as endocytosis [21,22], notochord development [23], neuromorphogenesis [24] and cell adhesion [25], but an involvement in cytokinesis has not been defined. We find that *Drosophila* Syndapin is ubiquitously expressed and is required for cytokinesis both in mitosis and male meiosis. Syndapin colocalizes with PI(4,5)P_2_ and directly binds to Anillin at the cleavage furrow, thus providing one component of the link between the plasma membrane and the contractile ring during cytokinesis. Either elevating or reducing the level of Syndapin induces cytokinesis defects with abnormal membrane behaviour, suggesting that Syndapin also regulates the dynamics of the cell cortex during cytokinesis. Finally, Syndapin's association with the furrow is prevented by phosphorylation; this reduces its membrane-binding affinity and deforming activity, suggesting a regulatory mechanism for cytoskeleton–membrane interaction during cytokinesis.

## 3. Results

### 3.1. *Drosophila* Syndapin localizes to the cleavage furrow during cytokinesis

To gain insight into the molecular nature of the coupling between the plasma membrane and the contractile ring in cytokinesis, we examined functions of a set of candidate proteins, the F-BAR proteins. We identified six F-BAR domain-containing proteins encoded in the *Drosophila* genome: Syndapin, Cip4, Nwk, FCHo/CG8176, Fps85D and NOSTRIN/CG42388 (see electronic supplementary material, figure S1a). Among these *Drosophila* F-BAR proteins, we examined the localization of Syndapin, Cip4, Nwk and FCHo/CG8176 by expressing them as GFP fusions in the cultured *Drosophila* cell line *D.Mel-2*. Syndapin::GFP localized to the cleavage furrow and the midbody throughout cytokinesis (see electronic supplementary material, figure S1b); Cip4::GFP localized to the midbody but only in late cytokinesis (see electronic supplementary material, figure S1c); and FCHo/CG8176 (see electronic supplementary material, figure S1d) and Nwk (not shown) were cytoplasmic. Syndapin formed discrete foci isotropically distributed on the cell cortex in metaphase but accumulated at the cleavage furrow upon anaphase onset (see electronic supplementary material, movie S1).

**Figure 1.**
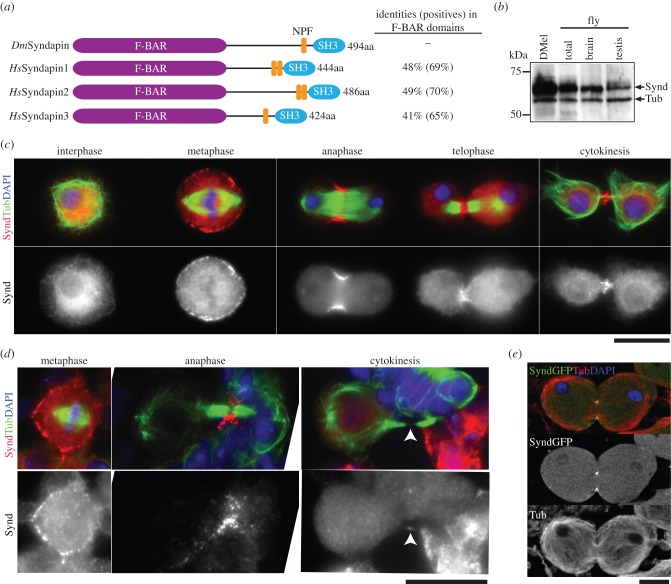
Drosophila Syndapin localizes to the cleavage furrow in cytokinesis. (*a*) Comparison of *Drosophila* Syndapin (*Dm*Syndapin) with human Syndapin (*Hs*Syndapin1, 2 and 3) showing principal domains and identities obtained by BLAST searches between primary sequences. (*b*) Immunoblot showing relative levels of Syndapin (Synd) and Tubulin (Tub) as loading control in extracts of *D.Mel-2* cells (DMel), total third instar larvae (total), third instar larval central nervous system (brain) and third instar larval testes (testis). (*c*) *D.Mel-2* cells immunostained to reveal endogenous Syndapin (red), Tubulin (green) and counterstained with DAPI to reveal DNA (blue). (*d*) Asymmetrically dividing neuroblasts stained to reveal Syndapin (red), Tubulin (green) and DNA (blue). (*e*) Primary spermatocytes in telophase of meiosis I expressing Syndapin::GFP (green) and stained to reveal Tubulin (red) and DNA (blue). Scale bars represent 10 µm.

These patterns of subcellular localization of F-BAR proteins led us to focus upon Syndapin because its association with the cleavage furrow suggested a role in cytokinesis. Syndapin is evolutionarily conserved and highly similar to its human orthologues, Syndapin 1, 2 and 3 (also called Pacsin 1, 2 and 3) (figure 1*a*). Syndapin contains both an F-BAR and an SH3 domain that are connected by a flexible linker containing an NPF (Asn-Pro-Phe) motif required for binding to EH domain proteins (EHDs) [26].

To confirm whether the localization profile of Syndapin::GFP corresponded to that of endogenous Syndapin, we raised an antibody against *Drosophila* Syndapin and analysed its localization by immunofluorescence microscopy. The anti-Syndapin antibody recognized a protein of around 65 kDa in both cultured *D.Mel-2* cells and fly extracts (figure 1*b*), which was depleted following Syndapin RNAi (see electronic supplementary material, figure S2a). Using immunofluorescence, localization of endogenous Syndapin was indistinguishable from Syndapin::GFP (figure 1*c*).

Syndapin is abundantly expressed in third instar larval brains (figure 1*b*), where it localizes to the cleavage furrow of neuroblasts during asymmetrical cell division (figure 1*d*). Syndapin is also expressed in testes but is slightly less abundant than in *D.Mel-2* cells or larval brains (figure 1*b*), and, despite several attempts, our antibody could not detect endogenous Syndapin by immunofluorescence microscopy. However, Syndapin::GFP expressed from a transgene could be detected at the cleavage furrow in primary spermatocytes (figure 1*e*; electronic supplementary material, movie S2). Thus, taken together, the localization of Syndapin is consistent with it having a role in cytokinesis during mitosis and male meiosis.

### 3.2. *Drosophila* Syndapin functions in cytokinesis

**Figure 2.**
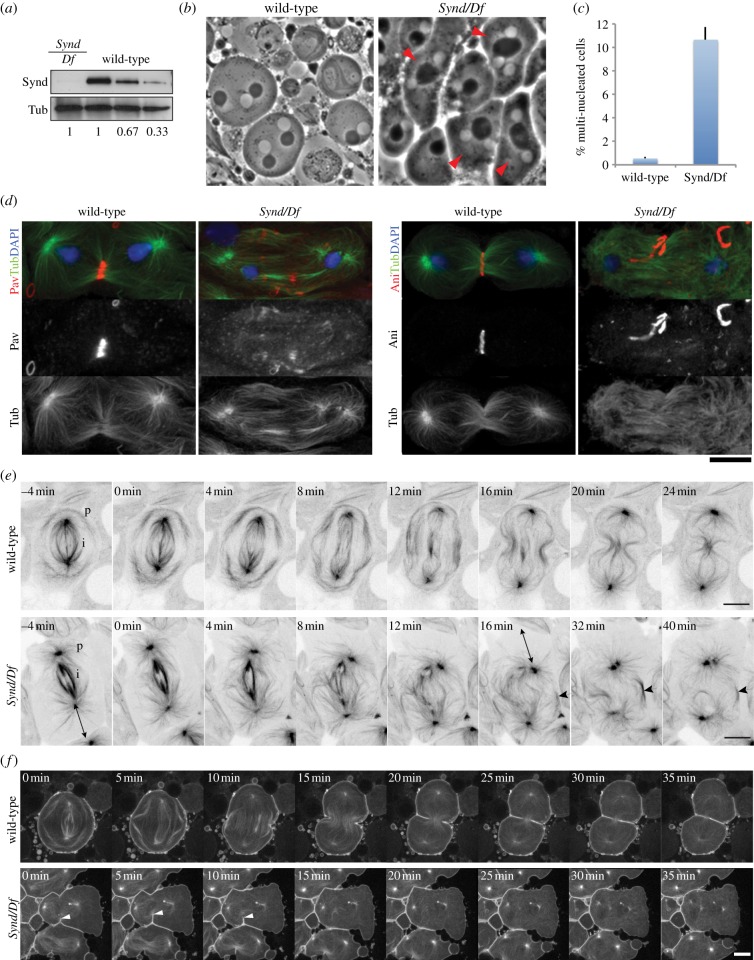
Syndapin is required for cytokinesis in male meiosis. (*a*) Immunoblot of extract from *Oregon R* (wild-type in a dilution series) and *Syndapin* mutant (*Synd/Df*) third instar larvae reveal expression levels of Syndapin (Synd) and Tubulin (Tub). (*b*) Phase contrast images of onion-stage spermatids in *Oregon R* (wild-type) and *Syndapin* mutant (*Synd/Df*) flies. Nuclei, white spheres; Nebenkern, black spheres. Multi-nulcleated cells are indicated (red arrows). (*c*) The *Syndapin* mutant (*Synd/Df*) showed 10.66% multi-nucleated spermatids in comparison with 0.55% in Oregon R (wild-type) flies. More than 60 and 400 cells were counted (*n* = 3) for quantifying the average proportion of multi-nucleate cells of *Synd/Df* and wild-type flies, respectively. Bars indicate SEs. (*d*) Spermatocytes in telophase of meiosis I from *Oregon R* (wild-type) and *Syndapin* mutant (*Synd/Df*) flies stained to reveal Pavarotti or Anillin (red), Tubulin (green) and DNA (blue). Pavarotti and Anillin were mislocalized in 38.5% (*n* = 13) and 41.7% (*n* = 12) of primary spermatocytes in the Syndapin mutant, while these defects were not observed in wild-type spermatids (*n* > 30). Scale bars represent 10 µm. (*e*) Time-lapse series of wild-type (upper) and *Syndapin* mutant (*Synd/Df*; lower) spermatocytes expressing tubulin::GFP in progression through cytokinesis. To highlight tubulin signals, inverted LUT images of tubulin::GFP (black) are shown. See Results for detail. p, Peripheral MTs; i, interior MTs. The extensive gap between cortex and spindle is indicated by double-headed arrows in Syndapin mutant spermatocytes. The arrows mark a rare example of peripheral central spindle bundles in the mutant that fails to ingress. Abnormal MT dynamics in cytokinesis were observed in 63.6% of *Syndapin* mutant spermatocytes (*n* = 11), while none of the wild-type spermatocytes (*n* = 21) showed the abnormalities. (*f*) Time-lapse series of wild-type (upper) and *Syndapin* mutant (*Synd/Df*; lower) spermatocytes expressing PLCδ-PH::GFP together with tubulin::GFP in progression through cytokinesis. The arrowheads mark asymmetrical ingression of the cleavage furrow in *Syndapin* mutant spermatocytes. Scale bars represent 10 µm.

To determine whether Syndapin functions in cytokinesis, we first generated a strong hypomorphic *Syndapin* mutant fly (*Synd^mut1^*) by imprecise excision of a P-element. The expression level of Syndapin was strongly reduced in *Synd^mut1^* (*Synd/Df*) compared with *Oregon R* (wild-type) flies (figure 2*a*). Almost all homozygous and hemizygous *Synd^mut1^* animals died at the third instar larval stage, consistent with the pleiotropic requirements for the protein in membrane trafficking that give Syndapin pivotal roles in *Drosophila* development. We then looked for cytokinesis defects in the male germ line in these mutants, because spermatogenesis offers a well-defined lineage independent of other developmental processes, making the testes an ideal tissue to study potential cell cycle roles of genes encoding proteins with other cellular functions. The primary spermatogonial cell that arises from a germ line stem cell undertakes four rounds of mitosis to form primary spermatocytes that, following an extended G2 phase, undertake the two meiotic divisions to produce spermatids. At the so-called ‘onion stage’, the post-meiotic spermatid cysts offer a highly effective way of assessing cytokinesis defects; in controls, these cysts contain cells having a single spherical nucleus and a mitochondrial aggregate, the Nebenkern and very few multi-nucleated cells (0.55%; figure 2*b,c*, wild-type). By contrast, we found that in *Synd^mut1^* flies, the proportion of multi-nucleated cells increased by almost 20-fold (10.66%; figure 2*b*,*c*, *Synd/Df*), indicating that Syndapin is required for cytokinesis during male meiosis.

We further characterized these cytokinesis defects by examining *Synd^mut1^* primary spermatocytes by immunofluorescence microscopy. During normal cytokinesis, the central spindle, an antiparallel array of microtubules (MTs), forms between segregated chromosomes and contains the furrowing signalling complex, centralspindlin (containing Pavarotti-KLP and RacGAP50C) at its midzone. In wild-type cells, the central spindle and its associated proteins were constricted by the contractile ring as the cleavage furrow ingressed and no cells showed abnormal cytoskeletal structures (figure 2*d*, wild-type). By contrast, *Synd^mut1^* mutant spermatocytes displayed a poorly organized central spindle, and a fragmented distribution of centralspindlin (Pavarotti) (38.5%, *n* = 13) and of the contractile ring component Anillin (41.7%, *n* = 12) (figure 2*d* *Synd*/*Df*).

To gain further insight into the cytokinesis defects in *Synd^mut1^*, we carried out time-lapse imaging of primary spermatocytes in transgenic flies expressing Tubulin::GFP [27] (figure 2*e*) and a membrane marker PLCδ-PH::GFP [28] (figure 2*f*). In wild-type spermatocytes, there are two distinct populations of astral MTs: peripheral MTs (p) and interior MTs (i) (figure 2*e*, wild-type, –4 min, p and i; electronic supplementary material, movie S3). In anaphase, the peripheral MTs and interior MTs formed cortical and central spindle MT bundles, respectively (figure 2*e*, wild-type, 0–12 min; electronic supplementary material, movie S3), and were finally pushed inwards and compacted by the ingressing cleavage furrow (figure 2*e*, wild-type, 16–24 min; electronic supplementary material, movie S3). In these processes, membrane invagination occurred symmetrically in more than 90% of cells (*n* = 21), and the overall cortical membrane behaviour was well coordinated with chromosome segregation and cell elongation (figure 2*f*, wild-type; electronic supplementary material, movie S5). By contrast, the peripheral MTs of *Synd^mut1^* spermatocytes were less robust (figure 2*e*, *Synd/Df*, –4 min; electronic supplementary material, movie S4), and the central spindle failed to form in an organized manner after anaphase onset (figure 2*e*, *Synd/Df*, 0–12 min; electronic supplementary material, movie S4). Unstable peripheral MT bundles occasionally formed but then disintegrated with failure of cleavage furrow ingression (figure 2*e*, *Synd/Df*, 16–40 min, arrowheads; electronic supplementary material, movie S4). The spindles in *Synd^mut1^* spermatocytes were often mispositioned with a gap between the centrosomes and cortex (figure 2*e*, *Synd/Df*, −4 min and 16 min, double-headed arrow; electronic supplementary material, movie S4). The abnormal MT dynamics in cytokinesis was observed in 63.6% of Syndapin mutant spermatocytes (*n* = 11), whereas none of the wild-type spermatocytes showed the abnormalities. Interestingly, ingression of the cleavage furrow in *Synd^mut1^* spermatocytes was often asymmetric, failing to progress to the interior of the cell and then regressing (85.7%, *n* = 7; figure 2*f*, *Synd/Df*, 0–10 min, arrowheads; electronic supplementary material, movie S6), suggesting an unstable interaction of cell membrane and cytokinetic machinery.

To confirm that Syndapin also functions in cytokinesis following mitosis, we analysed the effect of its depletion on cytokinesis in *D.Mel-2* cells. Syndapin was substantially depleted after 4 days of RNAi (see electronic supplementary material, figure S2*a*). Interestingly, more than 60% of Syndapin RNAi cells showed irregular cortical structures near the cleavage furrow (see electronic supplementary material, figure S2*b*,*c*) and about 10% of these failed cytokinesis (see electronic supplementary material, figure S2*d*,*e*). Thus, Syndapin is required for cytokinesis by contributing to the cortical behaviour in both mitosis and male meiosis.

### 3.3. Syndapin colocalizes with PI(4,5)P_2_ and Anillin at the cleavage furrow

**Figure 3.**
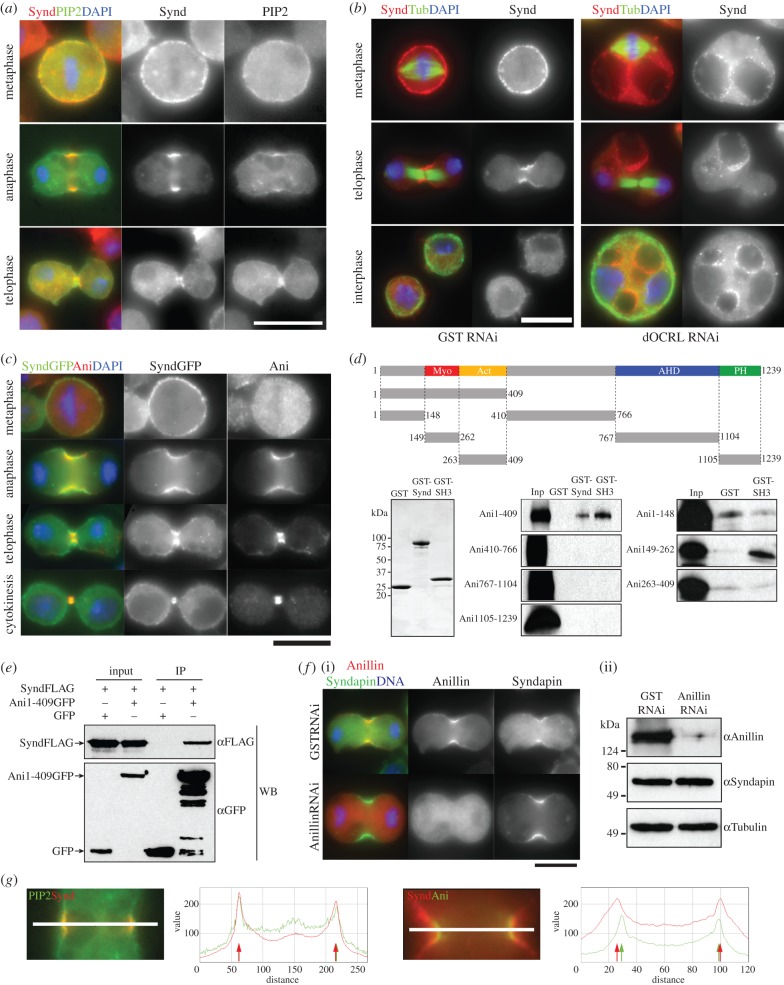
Syndapin colocalizes with PI(4,5)P_2_ and Anillin at the cleavage furrow. (*a*) *D.Mel-2* cells at the indicated mitotic stages expressing Tubby-GFP (green, to mark PI(4,5)P_2_) and stained to reveal Syndapin (red) and DNA (blue). (*b*) *D.Mel-2* cells following treatment with dsRNA targeted against inositol polyphosphate 5-phosphatase (dOCRL RNAi) or a control (GST RNAi). Staining reveals Syndapin (red), Tubulin (green) and DNA (blue). (*c*) *D.Mel-2* cells at successive stages of mitosis stained to reveal Syndapin (green), Anillin (red) and DNA (blue). (*d*) Segments of Anillin produced by coupled *in vitro* transcription/translation and used in binding assays with bacterially expressed GST-Syndapin (GST-Synd), GST-Syndapin-SH3 (GST-SH3) or control (GST) as indicated on lane headers of autoradiogram (Inp = Input). Segments of Anillin are indicated by each row as amino acid residues. (*e*) Co-immunoprecipitation experiment of FLAG-tagged Syndapin (SyndFLAG) co-transfected into *D.Mel-2* cells with either GFP or GFP-tagged Anillin N-terminal fragment (Ani1–409GFP). Immunoprecipitation using GFP-Trap was followed by immunoblotting using anti-GFP (αGFP) or anti-FLAG (αFLAG) antibodies. (*f*) Anillin is dispensable for Syndapin localization to the cleavage furrow in *D.Mel-2* cells. (i) *D.Mel-2* cells after 3 days RNAi of Anillin showing localization of Syndapin (green), Anillin (red) and DNA (blue). (ii) Depletion of Anillin after Anillin RNAi was confirmed by immunoblotting using anti-Anillin antibody (αAnillin) in comparison with the expression level of Syndapin (αSyndapin) and Tubulin (αTubulin) antibodies. (*g*) Relative distribution of Syndapin (red) with either PI(4,5)P_2_ or Anillin (green) at the cleavage furrow of a *D.Mel-2* cell. Intensity scan was carried out along the indicated line on the micrograph.

A previous study showed that *Drosophila* Syndapin associated with PI(4,5)P_2_ *in vitro* [23]. In yeast and in animal cells, PI(4,5)P_2_ localizes to the cleavage furrow and plays important regulatory roles in cytokinesis [29]. We therefore asked whether Syndapin also colocalized with PI(4,5)P_2_ during cytokinesis. To visualize the localization of PI(4,5)P_2_ in *D.Mel*-2 cells, we used a PI(4,5)P_2_ probe, Tubby-GFP, which has been successfully used in *Drosophila* cells in a previous study on cytokinesis [30]. As expected from previous findings, Syndapin and Tubby-GFP colocalized at the cleavage furrow (figure 3*a*), suggesting that Syndapin associated with PI(4,5)P_2_ during cytokinesis. We next asked whether interfering with phosphoinositide homeostasis might affect Syndapin localization. A PI(4,5)P_2_ phosphatase, OCRL (oculocerebrorenal syndrome of *L*owe), is required for cytokinesis in both *Drosophila* [30] and human cells [31]. OCRL knockdown in *Drosophila* S2 cells induces large intracellular giant vesicles enriched with PI(4,5)P_2_ and destabilizes the cleavage furrow [30]. Following OCRL RNAi, we found that Syndapin redistributed from the cell cortex to these intracellular giant vesicles (figure 3*b*). These results suggest that Syndapin localization responds to the phosphoinositide cycles associated with PI(4,5)P_2_ synthesis.

The above findings indicate that Syndapin associates with the plasma membrane at the cleavage furrow and raise the question as to how it might interact with the cytokinetic machinery. In our previous proteomic survey, Syndapin was found as one of Anillin interactors [32]. In fact, Syndapin colocalized with Anillin during cytokinesis in both cultured cells (figure 3*c*) and spermatocytes (see electronic supplementary material, figure S3*a*). To determine whether Syndapin and Anillin can interact, we tested this possibility by *in vitro* binding assay using GST-tagged Syndapin and *in vitro* translated ^35^S-labelled Anillin (figure 3*d*). This revealed a strong interaction between Syndapin's SH3 domain and the 149–262 amino acid segment of Anillin previously described as a myosin II binding domain [33], which contains two putative SH3-binding epitopes (-Pro-X-X-Pro-) (figure 3*d*). We confirmed this interaction by a co-immunoprecipitation assay between FLAG-tagged Syndapin and GFP-tagged Anillin upon expression of these proteins in cultured cells (figure 3*e*). In contrast to membrane lipids, Anillin was dispensable for Syndapin's localization to the cleavage furrow (figure 3*f*), suggesting that Syndapin may function by binding to membrane lipids, and so providing an interface for association with Anillin. Accordingly, high-resolution microscopy revealed that Syndapin, PI(4,5)P_2_ and Anillin have an overlapping distribution at the cleavage furrow, with Anillin lying at a more interior position than Syndapin (figure 3*g*).

### 3.4. Syndapin influences cortical dynamics in cytokinesis

**Figure 4.**
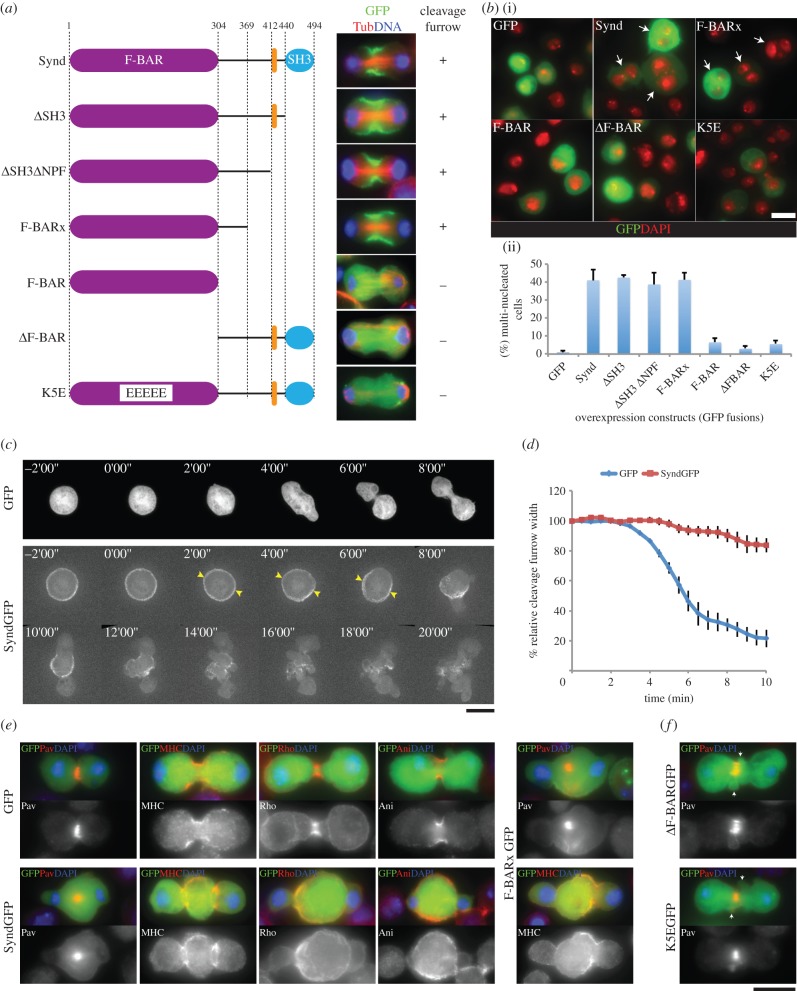
Syndapin controls cortical dynamics in cytokinesis. (*a*) Schematics of truncations or mutations of Syndapin tagged with GFP and expressed in *D.Mel-2* cells. Images showing localization of Syndapin fragments (green), Tubulin (red) and DNA (blue) in telophase cells. (*b*) Immunofluorescent images of cells transiently expressing indicated (i) Syndapin fragments for 3 days and (ii) quantification of the binucleated phenotype. More than 300 cells were counted (*n* = 3) for quantifying the average proportion of multi-nucleate cells. Bars indicate s.e. Arrows indicate binucleated cells. (*c*) Time-lapse series of *D.Mel-2* cells overexpressing either GFP (GFP) or Syndapin::GFP (SyndGFP) at the indicated intervals. Time 0'00’ is anaphase onset. Presumptive cleavage furrow sites are indicated in SyndGFP cells (yellow arrowheads). (*d*) Kinetics of the cleavage furrow showing cortical dynamics of cells overexpressing either GFP or Syndapin::GFP. Width of the cleavage furrow was measured from time-lapse series of cells overexpressing either GFP (GFP) or Syndapin::GFP (SyndGFP) and average width (*n* = 5 for each samples) were plotted over time as relative value to the starting width. Time point zero is anaphase onset. Bars indicate SEs. (*e*) Immunofluorescent images of *D.Mel-2* cells overexpressing either GFP (GFP), Syndapin::GFP (SyndGFP) or F-BARx::GFP (F-BARxGFP) (green) revealing Pavarotti (Pav), Myosin Heavy Chain (MHC), Rho1 (Rho), Anillin (Ani) (red) and DNA (blue). (*f*) Overexpression of cleavage furrow localization defective mutants (*Δ*F-BARGFP and K5EGFP) of Syndapin showing GFP-tagged Syndapin fragments (green), Pavarotti (red) and DNA (blue). Local blebs in the cleavage furrow are indicated (white arrows). Scale bars represent 10 µm.

To determine the functional contributions of lipid- and Anillin-binding properties of Syndapin in cytokinesis, we analysed localization of various segments of Syndapin to the cleavage furrow and their effect on cytokinesis upon overexpression in the presence of endogenous Syndapin. Deletion of Syndapin's SH3 domain (*Δ*SH3) and the NPF motif (*Δ*SH3*Δ*NPF) did not affect its localization (figure 4*a*), whereas deletion of the F-BAR domain (*Δ*F-BAR) or mutations in the F-BAR that disrupt binding to anionic lipids such as PI(4,5)P_2_ *in vitro* (K137E, K141E, K145E, K149E and K152E, which we refer to as K5E) [23] prevented localization to the cleavage furrow (figure 4*a*). Although necessary, the F-BAR domain of Syndapin alone was not sufficient for localization to the cleavage furrow (figure 4*a*, F-BAR), and an additional flanking 65 amino acids C-terminal to the F-BAR domain were required for it to localize to the cleavage furrow (figure 4*a*, F-BARx). Together these results concur with our above findings that Syndapin's colocalization with PI(4,5)P_2_ to the cleavage furrow requires its lipid-binding F-BAR domain.

We then examined the effects of expressing various GFP-tagged Syndapin fragments upon cytokinesis, including full-length Syndapin::GFP. We found that expression of Syndapin constructs that localized to the cleavage furrow had a disruptive effect on cytokinesis. Thus, for example, the full-length GFP-tagged protein induced an increase in multi-nucleated cells of around 40% in comparison with expression of GFP alone (figure 4*b*). Similarly, the other GFP-tagged furrow-localizing fragments (i.e. *Δ*SH3, *Δ*SH3*Δ*NPF and F-BARx) also induced a strong cytokinetic defect (figure 4*b*). We also found that expression of Syndapin::GFP in spermatocytes induced robust cytokinesis defects (see electronic supplementary material, figure S3*bc*). As expression of the *Δ*F-BAR mutant did not show a cytokinetic defect, the defect is unlikely to be owing to disruption of protein interactions with the SH3 but more likely to reflect loss of membrane-binding ability conferred by the F-BARx region.

We used time-lapse imaging to characterize better the cytokinesis defects caused by increased Syndapin expression. In control cells expressing GFP alone, the cleavage furrow started to ingress within 4 min after anaphase onset at 2.24 μm min^–1^ (s.e.m. = 0.15, *n* = 5) and achieved maximal ingression within 10 min (figure 4*c*, GFP; figure 4*d*; electronic supplementary material, movie S7). By contrast, the cleavage furrow of cells overexpressing exogenous Syndapin failed to ingress during anaphase (figure 4*c*, SyndGFP, 0.00–6.00; electronic supplementary material, movie S8). At telophase, the cytoplasm of overexpressing cells ballooned out around the nascent nuclei (figure 4*c*, SyndGFP, 8.00–10.00; electronic supplementary material, movie S8). At later times, there was more generalized blebbing that appeared over the entire cell surface (figure 4*c*, SyndGFP 12.00–20.00; electronic supplementary material, movie S8). In such cells, cleavage furrow ingression was slowed to 0.33 μm min^–1^ (s.e.m. = 0.13, *n* = 5; figure 4*d*). In cells expressing exogenous Syndapin or its F-BARx fragment, the central spindle formed properly, but contractile ring components likely to have membrane associations were mislocalized (figure 4*e*, SyndGFP and F-BARxGFP). By contrast, membrane-binding-deficient mutants did not prevent proper formation of the cleavage furrow, although some local blebbing was still observed (figure 4*f*, *Δ*F-BARGFP and K5EGFP).

### 3.5. Phosphoregulation of Syndapin in cytokinesis

**Figure 5.**
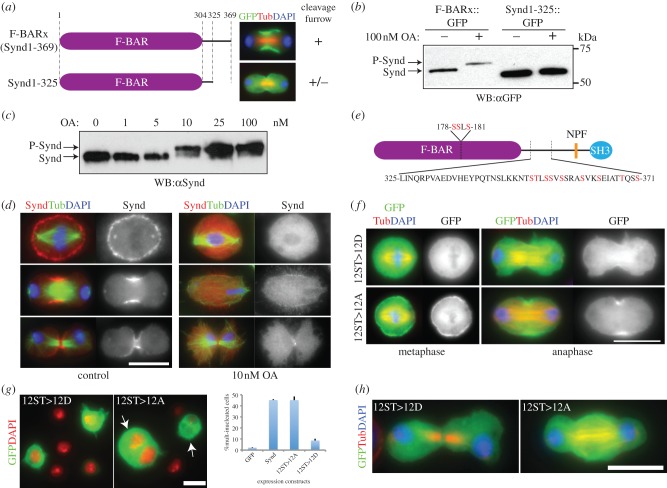
Syndapin localization and function is regulated by phosphorylation. (*a*) Immunofluorescent images showing localization of the GFP-tagged 1–369 amino acid fragment (F-BARx) and 1–325 amino acid fragment of Syndapin (Synd1–325) (green), Tubulin (red) and DNA (blue) at telophase. (*b*) Immunoblotting using anti-GFP antibody to detect cells expressing either F-BARx::GFP or Synd1–325::GFP untreated or treated with 100 nM OA for 2 h. (*c*) Immunoblot to detect Syndapin (Synd) or phosphorylated Syndapin (P-Synd) in total extracts of cells treated with indicated concentrations of OA for 2 h. (*d*) Localization of Syndapin (red), Tubulin (green) and DNA (blue) in cells treated with DMSO (control) or 10 nM OA for 2 h. (*e*) Phosphorylation sites identified by our phosphomapping study and in the published study [34] are shown in red. (*f*) Localization of GFP-tagged non-phosphorylatable (12ST>12A) or phosphomimetic (12ST>12D) forms of Syndapin (green), Tubulin (red) and DNA (blue). (*g*) *D.Mel-2* cells overexpressing indicated GFP-tagged Syndapin phosphomutants (green) and stained to show DNA (red). More than 300 cells were counted (*n* = 3) for quantifying the average proportion of multi-nucleate cells. Bars indicate s.e. (*h*) Overexpression of Syndapin phosphomutants showing GFP-tagged Syndapin mutants (green), Tubulin (red) and DNA (blue). Scale bars represent 10 µm.

The Synd F-BARx fragment (containing a 65-amino-acid C-terminal extension to the F-BAR domain) localized well to the cleavage furrow, a property that required residues 326–369 (figure 5*a*). The equivalent regions of fission yeast Cdc15p and budding yeast Hof1p have been reported to be phosphoregulated during cytokinesis [35,36], leading us to test the possibility that the 65-amino-acid extension to Syndapin's F-BAR domain is a regulatory region for membrane binding and has been evolutionarily retained from yeasts to metazoans. We found the mobility of Synd F-BARx (1–369 fragment), expressed in *D.Mel-2* cells, was shifted by treatment with the protein phosphatase inhibitor okadaic acid (OA), whereas mobility of the F-BAR+21 amino acids (Synd1–325 fragment) was unchanged (figure 5*b*). This mobility shift is thus likely to be owing to the phosphorylation of residues 326–369. OA differentially inhibits the two major families of serine/threonine phosphatases, PP1 and PP2A with respective IC_50_ s of 3 nM and 0.2–1 nM [37]. As we observed the mobility shift of Syndapin only when cells were treated with at least 10 nM OA, this implies that a PP1 family member may be responsible for dephosphorylation (figure 5*c*). Consistent with this, 10 nM OA resulted in loss of Syndapin from the cleavage furrow (figure 5*d*). Together these results suggest that Syndapin's localization to the cleavage furrow requires its dephosphorylation.

A previous phosphoproteome analysis of *Drosophila* embryos has identified multiple phosphorylation sites in Syndapin [34]. We independently employed mass spectrometry to map phosphorylation sites on affinity-purified Syndapin::PtA from OA-treated *D.Mel*-2 cells (electronic supplementary material, figure S4). Both approaches identified clusters of phosphorylation sites between residues 326 and 369 and in the F-BAR domain (figure 5*e*). To evaluate the biological significance of these phosphorylation sites, we mutated the 9 Ser (S) or Thr (T) target residues within amino acids 326–369 and 3 Ser residues in the F-BAR domain to either phosphomimetic (S or T to D (ST>D)) or non-phosphorylatable (S or T to A (ST>A)) amino acids and analysed both their subcellular localization and their effects upon cytokinesis. Neither the ST>A nor the ST>D mutants, with amino acid changes in all the 12 residues, underwent a mobility shift after OA treatment, confirming again that the electrophoretic shift of endogenous protein was likely to be owing to phosphorylation, and that the sites responsible for the shift had been identified by our phospho-mapping (see electronic supplementary material, figure S5a). Consistent with the mislocalization of Syndapin after 10 nM OA treatment, the phosphomimetic mutant in all 12 sites of the combined regions failed to localize to the furrow (figure 5*f*, 12ST>12D). By contrast, the non-phosphorylatable mutant could localize to the cleavage furrow (figure 5*f*, 12ST>12A), suggesting that folding of the protein was not perturbed by the mutations. Phosphomimetic mutants in either the three sites in the F-BAR domain alone or in the nine sites in the 326–369 segment still localized to the cleavage furrow (see electronic supplementary material, figure S5b). This strongly suggests that both the F-BAR and the extended region need to be dephosphorylated for cleavage furrow localization. Expression of the 12ST>12A mutant led to robust cytokinesis defects with ingression failure of the cleavage furrow, whereas expression of the 12ST>12D mutant had weak cytokinesis defects (figure 5*g*,*h*). This is a similar observation to that shown of the Synd K5E mutant, which also failed to localize to the cleavage furrow and gave no defect in cytokinesis (figure 4).

**Figure 6.**
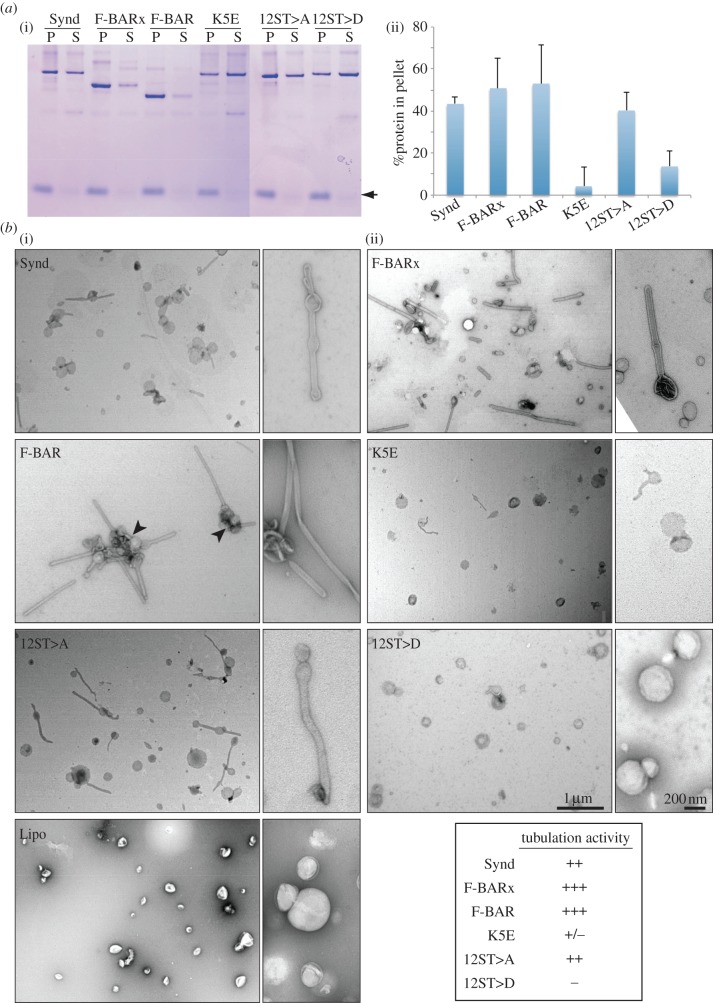
Membrane-binding and membrane-deforming activity of Syndapin is regulated by phosphorylation. (*a*) (i) Phosphorylation suppresses membrane-binding affinity of Syndapin. Liposome spin assay with purified recombinant Syndapin in full length (Synd), F-BAR domain +linker (F-BARx), F-BAR domain (F-BAR), K5E mutant (K5E), non-phosphorylatable mutant (12ST>A) and phosphomimetic mutant (12ST>D) was followed by SDS-PAGE. Black arrow indicates band corresponding to liposomes. (ii) The amount of proteins pelleting with and without liposome was quantified and the % of protein that pellets with liposomes was determined from three independent experiments, error bars±s.d.). (*b*) Phosphorylation inhibits liposome tubulation activity of Syndapin. Electron microscopic images of liposome mixed with various Syndapin fragments. (i) Lower magnification and (ii) higher magnification images are shown, nodes with tangled tubules formed by Syndapin F-BAR domains are indicated (F-BAR, arrowheads). Scale bars represent 1 µm and 200 nm for low magnification and high magnification images, respectively. Liposome tubulation activity of each fragments was categorized as strong (+++), mild (++), weak (+) and no activity (−) in the table.

To determine whether phosphorylation affects membrane binding, as suggested by the above *in vivo* experiments, we employed a liposome binding assay using purified fragments of Syndapin and its mutants. Syndapin constructs containing F-BAR domains efficiently bound and tubulated liposomes giving tubule diameters around 55 ± 11 nm (figure 6*a,b*, Synd, F-BARx and F-BAR). The full-length protein was less efficient in membrane binding and tubulation compared with F-BAR and F-BARx, as has previously been observed for mammalian Pacsin/Syndapin and reported to be owing to auto-inhibition [38,39]. By contrast, the lipid-binding mutant (K5E) bound to the liposomes much less efficiently and showed very little tubulation (figure 6*a*,*b*, K5E), although some rare but very narrow tubules are found. Interestingly, unlike tubules induced by either full-length Syndapin or the F-BARx fragment, tubules formed by the F-BAR domain alone frequently formed tangles (figure 6*b*, F-BAR, arrowheads). Importantly, the phosphomimetic mutant showed reduced membrane-binding and tubulating activity, while the non-phosphorylatable mutant bound and tubulated liposomes just as the wild-type protein (figure 6*a*,*b*, 12ST>A and 12ST>D). Together these results suggest that the phosphorylation status of Syndapin regulates its membrane-binding and deforming activity, and hence its role in cytokinesis.

## 4. Discussion

Here, we have provided the first compelling description of the requirements for an F-BAR protein in cytokinesis in animal cells. Our work does not exclude other F-BAR proteins from participating in cytokinesis, but it does show a positive role for Syndapin in cortical membrane dynamics at the cleavage furrow. Syndapin's localization to the cleavage furrow and its *in vitro* membrane binding and tubulation are regulated by phosphorylation. The defects in cytokinesis ensuing from phosphomimetic mutants imply that phosphorylation of Syndapin regulates cytokinesis by affecting its membrane association. However, this does not exclude a possible indirect effect whereby phosphorylation may influence the association between Syndapin's SH3 and F-BAR domains, as has been proposed to auto-inhibit its membrane association [38,39]. Our findings would suggest that auto-inhibition results in reduced membrane binding, and yet we do not see increased membrane binding and tubulation with the full-length 12ST>A mutant (figure 6*a,b*, compare Synd with F-BARx and 12ST>A). This implies that the major effect of phosphorylation is directly upon its membrane association. The phosphorylation of Syndapin could be a mechanism to prevent its premature association with the membrane at the cleavage furrow, as with phosphoregulation of Cdc15p during cytokinesis [36]. Thus, Syndapin joins the F-BAR proteins of *S. pombe* and *S. cerevisiae* (Cdc15p and Hof1p, respectively) as proteins that are also phosphoregulated during cytokinesis [35,36].

Syndapin's localization to the cleavage furrow requires its association with anionic lipids via its F-BAR domain (figure 3*a*,*b*). Syndapin also colocalizes with and directly binds to Anillin (figure 3*c*–*e*), but this interaction is dispensable for Syndapin localization (figure 3*f*). By contrast, Anillin is mislocalized during cytokinesis at least in primary spermatocytes of Syndapin mutants (figure 2*d*). Together these results led us to hypothesize that Syndapin may be a component of the coupling between the plasma membrane and the Anillin ring and hence the contractile ring. Alternatively, as Anillin itself has been proposed to have a role in linking the plasma membrane and the contractile ring [8,9], it is possible that Syndapin and Anillin share redundant function, and they may function cooperatively at the interface of plasma membrane and the contractile ring. Other candidate proteins for linking the contractile ring to the plasma membrane in cytokinesis are the C1 domain-containing MgcRacGAP of human cells [40] and the C2 domain-containing protein Inn1 of budding yeast [41]. Interestingly, Inn1 interacts with the F-BAR protein, Hof1p, and together they may cooperatively regulate membrane dynamics during cytokinesis in this organism. The *Drosophila* genome encodes several uncharacterized C2 domain-containing proteins, and it will be interesting to examine whether any of these proteins function cooperatively with Syndapin in cytokinesis. It is also possible that some of the other *Drosophila* F-BAR proteins (Cip4, Nwk, FCHo/CG8176, Fps85D and NOSTRIN/CG42388) function in cytokinesis. Such proteins could provide some functional redundancy to the molecular mechanism. Indeed, we cannot exclude the possibility that other molecular components can participate in safeguarding the linkage of the membrane to the contractile ring. The importance of such molecules might vary between tissues, thus accounting for the differences in severity of *Syndapin* phenotypes between different cell types.

Structure–function analyses demonstrated that expression of Syndapin fragments comprising the minimum segments required for the cleavage furrow localization (i.e. the F-BAR domain plus its C-terminal 65 amino acids) could induce dominant, strong cytokinesis defects (figure 4). Expression of exogenous Syndapin also induced similar abnormal cortical behaviour with furrow ingression failure and severe generalized blebbing. Surprisingly, despite the robustness of these cytokinesis defects, the localization of the major components of the central spindle (Pavarotti) was not affected, although contractile ring components were misplaced around the central part of the cell. By contrast, expression of Syndapin segments that fail to bind to anionic lipids and localize to the cleavage furrow (i.e. *Δ*FBAR and K5E) did not affect cytokinesis. These results suggest that Syndapin affects cortical dynamics during cytokinesis by directly associating with anionic lipids on the plasma membrane. The *S. pombe* F-BAR protein, Cdc15p, has roles in organizing membrane domains into lipid rafts as well as in the contractile ring formation [16]. Thus, it will be of future interest to determine whether Syndapin has an equivalent role in organizing membrane during cytokinesis in animal cells as a means of regulating cortical stiffness and dynamics [42,43].

Syndapin is required for synaptic vesicle recycling both in mice [44] and in flies (I.M.R. 2013, unpublished data), and an involvement in neuronal morphogenesis is regulated by developmentally controlled phosphorylation [45]. This raises the question of whether the functions of Syndapin in synaptic vesicle trafficking and other developmental processes might follow similar regulatory processes. The shape of a membrane can be described by the radius of curvature in two perpendicular arcs [46]. At the cleavage furrow, the radius of curvature along the axis of cell division will be positive, and perpendicular to this it will be negative. Similar curvatures will arise during vesicle recycling at the interface between the cap of a nascent vesicle and its parent membrane. The banana-shaped structure of F-BAR domains may make them ideal for associating with membrane in the context of such curvature, provided that all molecules orient in the one direction. An involvement of Syndapin in both cytokinesis and synaptic vesicle recycling would suggest that it can generate or stabilize varying degrees of positive curvature. When overexpressed in *D.Mel-2* cells, we sometime see narrow tubules decorated by Syndapin, and *in vitro* we observe tubules with approximate diameter of 55 nm. However, the diameter of positive curvature of a cleavage furrow will be at least one order of magnitude greater than this. Either Syndapin participates in forming smaller buds that become incorporated into the cleavage furrow or it indeed associates with membranes having larger diameters of curvature than may be suggested by the diameter of the concave face of its F-BAR domain. This latter possibility has some credibility because the extent of curvature will depend on the local membrane concentration of the F-BAR domain, and it would not be expected for the membrane to be saturated with the protein *in vivo* (otherwise, other membrane interacting proteins would be outcompeted). Thus, we might expect only narrow tubules to be formed either *in vitro* or, as a result of overexpression, *in vivo* when membrane sites could be saturated.

Several future challenges lay ahead before we can fully understand the regulation and roles of Syndapin in cytokinesis. Although the OA sensitivity of the protein phosphatase that dephosphorylates Syndapin suggests it is in the PP1 family, further studies are required to identify precisely the protein phosphatase(s) involved. Similarly, future studies will be necessary to identify the kinase(s) required for Syndapin's phosphoregulation. An understanding of Syndapin's precise cytokinetic role will be aided by more detailed description of its interacting partners. Although Syndapin interacts with Anillin, we still await full description of its functions in cytokinetic network. Only with this knowledge will we begin to understand how it might contribute to the coupling between the contractile ring and central spindle MTs underlying the cleavage furrow and the invaginating membrane.

## 5. Material and methods

### 5.1. Molecular biology

Expression constructs for GST-tagged Syndapin fragments were generated by cloning of the corresponding PCR products into pGEX4T-TEV (a gift from M. Mishima, University of Warwick). Gateway technology (Life Technologies) was used for all other cloning procedures as previously described [47]. Entry vectors were prepared by B-P recombination cloning of PCR products into pDONR221 vector, except for the entry vector for Tubby C-terminal (a kind gift from Amy Kiger, UCSD). Expression constructs for *D.Mel*-2 cells (GFP-, FLAG- or Protein A-tagged proteins with constitutive actin 5c promoter) or flies (GFP-tagged proteins with ubiquitin promoter) were created by L-R recombination cloning of the entry clones with corresponding destination vectors.

### 5.2. Cell culture, RNAi and DNA transfection

*D.Mel-2* cells were grown in serum-free Express Five SFM medium (Life Technologies) supplemented with 2 mM l-glutamine and 1% Penicillin-Streptomycin at 25°C. For RNAi treatment, double-stranded RNA (dsRNA) was prepared using the T7 RiboMAX Express RNAi System (Promega) and 20 µg was used for transfecting 1 × 10^6^ cells in six well plates with TransFast transfection reagent (Promega) following manufacturer's instructions. The RNAi was induced for 3–5 days and used for further phenotypic analyses. To transfect *D.Mel*-2 cells, 3 × 10^6^ cells in six well plates were transfected with 1.5 µg expression plasmid using FuGENE (Promega). To examine the consequences of the expression of Syndapin or its fragments, cells were collected after 3 days of the transfection for phenotypic analyses. For generating Blasticidin-resistant stable cell lines, 0.3 µg pCoBlast (Life Technologies) were co-transfected together with expression plasmids and transgenic cells were selected by adding Blasticidin (Life Technologies) to the medium at the final concentration of 50 µg ml^−1^. The same process also eliminated cells in which expression levels resulted in lethality.

### 5.3. Fly stocks and genetics

Flies were raised on standard cornmeal medium at 25°C. The Syndapin hypomorph mutant was generated by *P*-element excision of the *P{EP}Synd^EP877^* obtained from Bloomington. We recovered a severe hypomorphic allele (*Synd^mut1^*) in which a 536 bp deletion was found extending to the right of the insertion site of EP(3)0877. The deletion removes approximately half of the first exon and part of the first intron. The deficiency *Df*(*3R*)*BSC43* which uncovers the Syndapin gene was obtained from Bloomington. The *β-Tubulin::GFP* used in the immunofluorescent microscopy was described previously [27]. The transgenic flies expressing PLCδ-PH-GFP (a gift from Julie Brill, University of Toronto) was described previously [28]. Transgenic fly lines of Ub-Syndapin::GFP and UASp-Syndapin::GFP were generated by BestGene, Inc.

### 5.4. Antibodies

Anti-Syndapin antibody was raised in rabbits against purified His-tagged Syndapin F-BAR domain. Serum production was performed by Harlan Laboratories. Other antibodies used in this study were a mouse monoclonal anti-tubulin (clone DM1A; Sigma-Aldrich), rabbit anti-Pav-KLP [48], rabbit anti-Anillin (a gift from Julie Brill, University of Toronto), rabbit anti-Myosin Heavy Chain (MHC) (a gift from Roger Karess, Institut Jaques Monod), mouse anti-Rho antibody (clone p1D9, Developmental Studies Hybridoma Bank), mouse anti-GFP antibody (clones 7.1 and 13, Roche Applied Science) Peroxidase-ChromPure anti–rabbit IgG and anti-mouse IgG (Jackson ImmunoResearch Laboratories, Inc.). Alexa Fluor–conjugated secondary antibodies were purchased from Life Technologies.

### 5.5. Immunostaining of *D.Mel-2* cells, spermatocytes and neuroblast

For immunostaining of *D.Mel-2*, cells grown on coverslips were fixed with a fixative (4% formaldehyde, 60 mM PIPES, 30 mM Hepes, 10 mM EGTA and 4mM MgSO4, pH 6.8) for 12 min. After washing with PBS, the cells were permeabilized and blocked with PBS containing 0.5% Triton X-100 and 3% BSA for 1 h. The samples were then incubated with primary antibodies diluted in PBSTB (PBS containing 0.1% Triton X-100 and 1% BSA) overnight at 4°C in a humid chamber. After washing with PBSTB, the cells were incubated with secondary antibodies diluted in PBSTB for 4 h at room temperature. Then, the cells are washed with PBSTB and mounted in Vectashield with DAPI. For immunostaining of spermatocytes, testes were dissected from third instar larvae in PBS, squashed in PBS containing 5% glycerol and quickly frozen in liquid nitrogen. The samples were then fixed using ice-cold methanol for 10 min and permeabilized in PBS with 0.5% Triton X-100 for 30 s. After washing with PBS for 10 min, the samples were blocked with PBS containing 0.1% Triton X-100 and 1% BSA. Incubations with primary antibodies were performed overnight at 4°C in a humid chamber. Preparations were then incubated with secondary antibodies at room temperature for 4 h. Samples were washed with PBS and mounted in Vectashield with DAPI. Neuroblast immunostaining was performed as previously described [49]. Dilution of antibodies were as follows: anti-Syndapin (1 : 1000 for *D.Mel-2* and 1 : 100 for spermatocytes and neuroblasts), anti-Pav-KLP (1 : 750 for *D.Mel-2* and 1 : 75 for spermatocytes and neuroblasts), anti-Anillin (1 : 100), anti-MHC (1 : 1000), anti-Rho (1 : 50) and Alexa Fluor-conjugated secondary antibodies (1 : 500 for *D.Mel-2* and 1 : 50 for neuroblasts and spermatocytes).

### 5.6. Microscopy

For phase-contrast imaging of onion-stage cysts, testes were dissected in 0.7% NaCl solution and gently squashed under a coverslip until the appropriate degree of flattening was attained. Specimens were screened for intact cysts of primary spermatocytes using phase contrast on a Nikon Microphot-FX microscope at low magnification [25], and the morphology and number of cells in those cysts were analysed. Images were acquired with an AxioCam camera with AxioVision software (Carl Zeiss, Inc.). Fixed *D.Mel*-2 cells were visualized using an Axiovert 200 fluorescence microscope (Carl Zeiss, Inc.) with a 100× NA 1.4 objective lens, and images were acquired using a Coolsnap HQ camera (Photometrics) and Metamorph software (MDS Analytical Technologies). Fixed testes were visualized on a confocal microscope (LSM510 Meta; Carl Zeiss, Inc.) with 100× NA 1.4 objective lens. For time-lapse imaging of *D.Mel-2* cells expressing Syndapin::GFP and GFP, cells were maintained in open chambers at 25°C and images were acquired on a Zeiss Axiovert 200 microscope fitted with RSIII spinning disc confocal unit using Volocity software (PerkinElmer Life Sciences). Ten optical sections were captured at 30 s intervals with a 100× NA 1.4 lens and a 2 × 2 bin. Time-lapse imaging analyses of primary spermatocytes and neuroblasts were performed using a 100× NA 1.4 objective lens on a fluorescence microscope outfitted with excitation, emission and neutral density filter wheels (Prior Scientific), and a *z*-axis focus drive (PIFOC; Physik Instruments). Samples were maintained at a constant temperature of 25°C throughout filming. Images were acquired using a Coolsnap HQ camera and Metamorph software. All images were analysed using ImageJ (National Institutes of Health) and processed in Photoshop (Adobe).

### 5.7. *In vitro* protein binding assay and co-immunoprecipitation assay

The GST-tagged Syndapin fragments were purified using glutathione sepharose 4B according to the manufacturer's instructions (GE Healthcare). [35S]-methionine-labelled Anillin fragments were prepared from the corresponding PCR fragments amplified with primers harbouring a T7 promoter, and then transcribed and translated *in vitro* using the TnT T7 Quick Coupled Transcription/Translation Systems (Promega) in the presence of [35S]-methionine. Generally, 25 µl of glutathione sepharose beads containing purified GST-Syndapin fragments were mixed with 5 µl of [35S]-methionine-labelled Anillin fragments and 300 µl of NET-N+ buffer (50 mM Tris–HCl, pH 7.4, 150 mM NaCl, 5 mM EDTA, 0.5% NP-40 and a cocktail of proteinase inhibitors commercially available from Roche), and incubated on ice for 30 min with periodic agitation. The mixture was then washed five times by adding 500 µl of NET-N + buffer followed by centrifugation at 1500 r.p.m. in a benchtop centrifuge for 1 min. Beads were resuspended in 25 µl of 2× SDS sample buffer and typically one-fifth of the mixture (10 µl) was loaded on 4–20% tris-glycine gel (Invitrogen). Proteins were then transferred onto a nitrocellulose membrane using the iBlot dry blotting system (Invitrogen) and exposed to X-ray films at −80°C.

Co-immunoprecipitation assay of Syndapin and Anillin was performed using GFP-Trap (ChromoTek). In short, *D.Mel-2* cells were transfected with pAc-Syndapin::FLAG and pAc-Anillin1–409::GFP for 3 days, and total cell extract was used for co-immunoprecipitation analyses following the manufacturer's instructions.

### 5.8. Liposome co-sedimentation and *in vitro* tubulation assays

For protein-membrane-binding experiments, 200 nm liposomes were made by pore extrusion. Liposomes were composed of 99% Folch brain-derived lipids—1 : 1 mixture of Avanti Polar Lipids, (141101) and Sigma Folch (B1502) and 1% PI(4,5)P_2_. Lipid components were mixed in 9 : 1 chloroform : methanol, dried in glass tubes by argon gas, rehydrated in buffer (150 mM NaCl, 20 mM HEPES pH 7.4 and 2.5 mM DTT) to a final concentration of 1 mg ml^−1^ and filtered through Whatman 0.2 µm diameter polycarbonate filters. Syndapin proteins used in the assay were expressed and purified as GST fusions, and the GST tag was removed by TEV protease. For lipid co-sedimentation assays, 10 µM protein was incubated with 5 µl of 0.5 mg ml^−1^ liposomes in a total volume of 40 µl for 30 min at room temperature, and then spun down in a benchtop ultracentrifuge (Optima TL Ultracentrifuge) for 15 min at 80 000 r.p.m. (rotor TLA100). The supernatant was separated from the pellet, both were resuspended in sample buffer, and samples were boiled and run on SDS-PAGE gels. For *in vitro* tubulation assays, protein was incubated as above at room temperature for 15–30 min and pipetted onto glow-discharged carbon-coated copper TEM grids (Agar brand) for approximately 1 min. Grids were negatively stained with 2% uranyl acetate for 60 s, washed in water briefly and dried by blotting. Samples were examined on a PW6010/20 EM2055 transmission electron microscope (Philips).

### 5.9. Site-directed mutagenesis

Site-directed mutagenesis of Syndapin cDNA to express Syndapin lipid-binding-deficient mutant (K5E) and phosphomutants (S/T>D and S/T>A) was performed using QuikChange XL Site-Directed Mutagenesis Kit (Agilent Technologies) following manufacturer's instructions. Oligonucleotide used in the site-directed mutagenesis is shown in the electronic supplementary material, table S1.

### 5.10. Phospho-mapping of Syndapin

*D.Mel-2* cells stably expressing protein A-tagged Syndapin was treated with either 100 nM OA or DMSO, and phosphorylated or unphosphorylated form of Syndapin was purified using affinity purification as described previously [50] but in the presence of phosphatase inhibitors (PhosSTOP, Roche) in all the buffers used. The purified samples were separated on a 2D SDS-PAGE gel and stained with Coomassie blue. Excised bands were digested overnight with trypsin and the peptide mixtures were subsequently analysed by mass spectrometry. In order to determine sites of phosphorylation, samples were analysed on a 4000 QTrap instrument and the MIDAS approach was applied, essentially as described previously [51,52].

## Supplementary Material

Supplementary Information

## Supplementary Material

Supplementary Figure S1

## Supplementary Material

Supplementary Figure S2

## Supplementary Material

Supplementary Figure S3

## Supplementary Material

Supplementary Figure S4

## Supplementary Material

Supplementary Figure S5
