# Supplementary material for: Drosophila F-BAR protein Syndapin contributes to coupling the plasma membrane and contractile ring in cytokinesis
Source: Open Biol. 2013 Aug;3(8):130081. doi: 10.1098/rsob.130081 (PMC3758542; doi:10.1098/rsob.130081)
Supplement: Supplementary Information [file rsob130081-s1.docx]

***SUPPLEMENTARY INFORMATION***

***Supplementary Table 1***

**Syndapin non-phosphorylatbale mutant**

SyndS350A_S351A gcctgaagaagaacaccgccgccttgagcagtgtcagc

SyndS350A_S351A_antisense gctgacactgctcaaggcggcggtgttcttcttcaggc

SyndS356A_S360A_S363A ccttgagcagtgtcgccagcagagcggctgtgaaggccgaaatagcgacca

SyndS356A_S360A_S363A_antisense tggtcgctatttcggccttcacagccgctctgctggcgacactgctcaagg

SyndS371A cgaccacgcaatccgcagtcaccacatcg

SyndS371A_antisense cgatgtggtgactgcggattgcgtggtcg

**Syndapin phosphomimetic mutant**

SyndS350D_S351D aacagcctgaagaagaacaccgacgacttgagcagtgtcagcagcag

SyndS350D_S351D_antisense ctgctgctgacactgctcaagtcgtcggtgttcttcttcaggctgtt

SyndS356D_S360D_S363D caccttgagcagtgtcgacagcagagcggatgtgaaggacgaaatagcgaccacg

SyndS356D_S360D_S363D_antisense cgtggtcgctatttcgtccttcacatccgctctgctgtcgacactgctcaaggtg

SyndS371D atagcgaccacgcaatccgatgtcaccacatcggaagcc

SyndS371D_antisense ggcttccgatgtggtgacatcggattgcgtggtcgctat

SyndS181A ccgatagctcgttggcgccggatcaggtg

SyndS181A_antisense cacctgatccggcgccaacgagctatcgg

SyndS181D gccaatgccgatagctcgttggatccggatcaggtgaagaaaatg

SyndS181D_antisense cattttcttcacctgatccggatccaacgagctatcggcattggc

SyndS353354D agaagaacaccgacgacttggacgatgtcgacagcagagcggatg

SyndS353354D_antisense catccgctctgctgtcgacatcgtccaagtcgtcggtgttcttct

SyndT368D gtgaaggacgaaatagcgaccgatcaatccgatgtcaccacatcg

SyndT368D_antisense cgatgtggtgacatcggattgatcggtcgctatttcgtccttcac

SyndS353354A gaacaccgccgccttggccgctgtcgccagcagagcg

SyndS353354A_antisense cgctctgctggcgacagcggccaaggcggcggtgttc

SyndT368A ccgaaatagcgaccgcgcaatccgcagtc

SyndT368A_antisense gactgcggattgcgcggtcgctatttcgg

SyndS178D caggaacgcaatgccaatgccgatgacgatttggatccggatcaggtgaag

SyndS178D_antisense cttcacctgatccggatccaaatcgtcatcggcattggcattgcgttcctg

SyndS178A cgcaatgccaatgccgatgccgcgttggcgccg

SyndS178A_antisense cggcgccaacgcggcatcggcattggcattgcg

**Syndapin K5E mutant**

SyndK137E_S cctggaggatctgttcgagaaggcccagaaacc

SyndK137E_AS ggtttctgggccttctcgaacagatcctccagg

Synd K141E_K145E_S gaaggcccaggagccctgggccgagctgctggc

Synd K141E_K145E_AS gccagcagctcggcccagggctcctgggccttc

Synd K149E_K152E S ggccgagctgctggcagaggtcgaggaggccaaag

Synd K149E_K152E AS ctttggcctcctcgacctctgccagcagctcggcc

***Supplementary Figure S1. Drosophila F-BAR proteins*** (a) Schematic illustrations of 6 *Drosophila* F-BAR proteins, Syndapin(CG33094), Cip4(CG15015), Nwk(CG4684), FCHo(CG8176), Fps85D(CG8874) and NOSTRIN(CG42388). (b) *D.Mel-2* cells expressing a stable Syndapin::GFP transgene (green) and stained to reveal Tubulin (red) and DNA (blue). (c) *D.Mel-2* cells expressing a stable Cip4::GFP transgene (green) and stained to reveal Tubulin (red) and DNA. (d) *D.Mel-2* cells expressing a stable FCHo::GFP transgene (green) and stained to reveal Tubulin (red) and DNA.

***Supplementary Figure S2. Syndapin RNAi induces mild cytokinesis defects with abnormal cortical structures*** (a) Immunoblot of *D.Mel-2* cells following control (GST) or Syndapin (Synd) RNAi stained to reveal Syndapin (Synd) and Tubulin (Tub) as loading control. (b) Depletion of Syndapin induces abnormal cortex in cytokinesis. Localisation of Syndapin (Synd, green), Tubulin (Tub, red) and DAPI (DNA, blue) in telophase/cytokinesis cells after control (GST RNAi) or *Syndapin* (Synd RNAi) RNAi are shown. Abnormal cortical bulges at the cleavage furrow in induced after Syndapn RNAi are indicated (white arrows). (c) Telophase / cytokinesis cells with abnormal cortical structures account for 15.3% (n>50, N=3) forcontrol (GST) and 64.4% (n>50, N=3) for Syndapin (Synd) RNAi treated cells. Error bars indicate SEs. (d) Binucleate *D.Mel-2* cell (arrow) formed after Syndapin RNAi treatment (right) but not control RNAi (left). Tubulin, red; DNA, blue. (e) Binucleate cells account for 2.4% (n>350, N=3) or 5.80±0.44% (n>400, N=3) for control (GST) RNAi and *Syndapin* (Synd) RNAi treated cells, respectively. Error bars indicate SEs.

***Supplementary Figure S3. Syndapin localisation and overexpression phenotype in male meiotic cytokinesis*** (a) spermatocytes showing localisation of Syndapin::GFP (green), Anillin (red) and DNA (blue). (b) Phase contrast images of onion stage spermatids in Syndapin-overexpressing flies. Binucleate cells in wild-type (*Oregon R*) and two different transgenic flies of Syndapin::GFP (3M and 1M) are quantified by counting more than 80 spermatids. (c) Immunofluorescent images of spermatocytes overexpresing Syndapin::GFP showing Anillin (red), Tubulin (green) and DNA (blue). Scale bar, 10μm.

***Supplementary Figure S4. MS/MS spectra showing the major diagnostic fragment ions enabling phosphorylation site determination.***

***Supplementary Figure S5. Characterization of Syndapin phosphomutants*** (a) Immunoblot using anti-GFP antibody on extracts of *D.Mel-2* cells expressing GFP fusions of either wild-type (Synd::GFP), non-phosphorylatable (12ST>12A::GFP) or phosphomimetic (12ST>12D::GFP) that were either untreated or treated with Okadaic acid (OA). (b) Localization of GFP-tagged non-phosphorylatable (ST>A) or phosphomimetic (ST>D) mutants of Syndapin in either the F-BAR (F-BAR, 3ST>3A and 3ST>3D), medial region (medial, 9ST>9A and 9ST>9D) or the two regions together (F-BAR+medial, 12ST>12A and 12ST>12D). GFP-fusion proteins (green), Tubulin (red) and DNA (blue).

***Supplementary movie S1 Syndapin::GFP in D.Mel-2 cells***

***Supplementary movie S2 Syndapin::GFP in primary spermatocytes***

***Supplementary movie S3 Tubulin::GFP in primary spermatocyte of wild type flies***

***Supplementary movie S4 Tubulin-GFP in primary spermatocyte of Syndapin mutant flies***

***Supplementary movie S5 PLCdPH::GFP in primary spermatocyte of wild type flies***

***Supplementary movie S6 PLCdPH::GFP in primary spermatocyte of Syndapin mutant flies***

***Supplementary movie S7 GFP overexpression in D.Mel-2 cells***

***Supplementary movie S8 Syndapin::GFP overexpression in D.Mel-2 cells***
